# Mechanism of Grain Refinement in 3D-Printed AlSi10Mg Alloy Subjected to Severe Plastic Deformation

**DOI:** 10.3390/ma17164098

**Published:** 2024-08-19

**Authors:** Przemysław Snopiński, Ondřej Hilšer

**Affiliations:** 1 Department of Engineering Materials and Biomaterials, Silesian University of Technology, 18A Konarskiego Street, 44-100 Gliwice, Poland; 2 Faculty of Mechanical Engineering, VSB-TU Ostrava, 17. Listopadu 2172/15, 708 00 Ostrava, Czech Republic; ondrej.hilser@vsb.cz

**Keywords:** AlSi10Mg, ECAP, microstructure, grain refinement, EBSD, TKD

## Abstract

In this article, the evolution of microstructural characteristics of selectively laser-melted AlSi10Mg alloy subjected to equal channel angular pressing (ECAP) is investigated. The microstructures were analyzed in detail using scanning electron microscopy (SEM), electron backscatter diffraction (EBSD), transmission Kikuchi diffraction (TKD), and transmission electron microscopy (TEM). A heterogeneous ultrafine-grained microstructure was produced after one ECAP pass at 100 °C. This microstructure was composed of Al/Si cells and sub-micrometer grains. The grains were refined by conventional dislocation processes; however, evidence of dynamic recrystallization was also documented. Furthermore, it was revealed that the Al/Si cells contribute significantly to grain refinement. EBSD/TKD investigations showed that cell misorientation increased after ECAP processing, resulting in an increased fraction of grains with very low misorientation angles.

## 1. Introduction

Grain refinement in metallic materials has been an area of intensive research for several decades. This is because fine-grain materials exhibit numerous desirable properties, including improved formability and superplasticity, along with increased strength and ductility. This enhancement is primarily attributed to the Hall–Petch effect [1,2,3], a relationship that correlates grain size with material yield strength.

The Hall–Petch effect, named after its discoverers Hall and Petch, states that a material’s yield strength increases as the grain diameter decreases. This occurs because grain boundaries hinder dislocation movement, which impedes plastic deformation and enhances strength. As grain size decreases, the number of grain boundaries increases, further restricting dislocation movement and thus increasing strength [4].

Grain refinement can occur during and after the solidification process [5], as well as during thermomechanical processing [6] or cold plastic deformation [7]. However, conventional methods are only capable of refining grains down to the sub-micrometer scale. These limitations have led to a paradigm shift toward synthesizing nanostructured materials and developing alternative processing techniques. Among these, the severe plastic deformation (SPD) technique stands out [8]. This process involves imposing extremely high strains at relatively low temperatures, effectively producing materials with ultrafine and/or nano-grain structures.

Grain refinement is a crucial technique for enhancing the properties of aluminum alloys, leading to extensive research into Severe Plastic Deformation (SPD) post-processing methods in recent years [9,10,11,12]. Among the various SPD techniques available, such as multidirectional forging (MDF), high-pressure torsion (HPT) [13], and accumulative roll bonding (ARB) [14], equal-channel angular pressing (ECAP) [15] has garnered the most significant attention from the scientific community [10,16,17]. Numerous studies have systematically examined the influence of ECAP on the grain refinement and mechanical properties of various aluminum alloys [6,18,19,20]. In this technique, a billet is pressed through a die composed of two identical channels that intersect at a specific angle. During plastic deformation, the billet experiences severe shear deformation while maintaining its initial cross-sectional area. The pressing procedure can be repeated multiple times, with each repetition further refining the grain size and consequently improving the mechanical properties [21].

In conventional single-phase materials, grain refinement occurs through the break-up of very large and uniform grains. Dislocation theory suggests that during the initial stages of deformation, a substantial dislocation accumulation occurs, resulting in the creation of an intragranular structure composed of cells with thick walls with low misorientation angles. With increasing strain, these cell walls become thinner and transform into grain boundaries. Ultimately, this process leads to the development of ultrafine grains characterized by high-angle, non-equilibrium grain boundaries (GBs) [22,23]. In contrast, heterostructure (HS) materials exhibit distinct deformation mechanisms. According to the Taylor work-hardening model, geometrically necessary dislocations (GNDs) accumulate near heterozones (interfaces), while statistically stored dislocations (SSDs) remain confined to areas of minimal local energy [24,25,26]. As a result, various dislocation structures, such as dislocation cells, dislocation walls, and microbands, can form. Notably, GNDs play a crucial role in grain refinement by serving as obstacles to mobile dislocations, thereby promoting grain subdivision [27]. Therefore, thoroughly understanding dislocation structures and their associated phenomena is essential for investigating the grain refinement process in HS materials. This knowledge is also crucial for advancing the development of additively manufactured HS materials with optimized mechanical properties.

The HS materials are usually produced via the thermomechanical route, which involves several deformation and annealing cycles [28]. However, a heterogeneous microstructure can also be produced by laser powder-bed fusion (L-PBF) [29]. L-PBF is a widely popular metal additive manufacturing (AM) technique that fabricates parts by selectively melting thin continuous layers of metal powders [30,31]. L-PBF involves rapid and spatially variable heating, melting, cooling, and solidification cycles of the printed material. During this process, the rapid solidification of the liquid–solid phase transformation happens at exceptionally high cooling rates, often surpassing 10^6^ K s ^−1^ [32]. As a result, non-equilibrium solidification microstructures develop under conditions of significant undercooling and rapid grain growth. This leads to enhanced solute solid solubility, a much finer grain size compared to traditional processing methods, and decreased element segregation [33,34].

Recently, several research works have been conducted to investigate the effects of ECAP and HPT on the microstructure of aluminum alloys fabricated by L-PBF. It was shown that exceptional mechanical properties can be achieved when the microstructure is refined down to the ultrafine-grained regime. For example, Al-Zubaydi processed an Al–9%Si–3%Cu using the HPT method and reported a tensile strength of 700 MPa [35]. Muñoz et al. post-processed a hypoeutectic AlSi11Cu alloy using the ECAP method, and reported a tensile strength of ~600 MPa [36]. In another study, Hosseinzadeh used the same method to post-process the L-PBF AlSi12 alloy. They reported a tensile strength of ~500 MPa after four ECAP passes [37].

Despite extensive research on the mechanical properties resulting from SPD treatments [38,39,40,41,42], there is a considerable shortage of studies dedicated to a detailed analysis of grain refinement mechanisms. Understanding the complexity of grain refinement is crucial because it directly affects the mechanical behavior and performance of SPD-treated materials. However, the literature mainly focuses on the final results of SPD processing, with little attention to the fundamental processes that drive the reduction in grain size and the subsequent formation of stable and refined microstructures. This discrepancy in the current research landscape highlights the need for a comprehensive investigation of grain refinement phenomena during the SPD. Such an analysis is essential to optimize SPD techniques and tailor the microstructure characteristics of materials to achieve the desired mechanical properties.

The primary goal of this research is to analyze the grain refinement mechanisms in AlSi10Mg alloy processed by ECAP using advanced electron microscopy techniques. By thoroughly characterizing the microstructural changes, the study seeks to develop a comprehensive model of grain refinement that can be applied to enhance the properties of AlSi10Mg and similar L-PBF alloys. The research also aims to identify the specific role of Al/Si cells in the grain refinement process, which could lead to more targeted and effective post-processing treatments for additively manufactured materials.

In this study, we use state-of-the-art electron microscopy methods, including SEM, EBSD, TKD, and TEM, providing a detailed analysis of the microstructural evolution, and offering insights that are often missed by conventional characterization techniques. In addition, unlike many studies that focus on homogeneous materials, this research investigates the behavior of a heterogeneous microstructure formed by the additive manufacturing process, specifically examining how these unique structures respond to severe plastic deformation. Furthermore, the study aims to go beyond descriptive analysis by developing a predictive model of grain refinement, which can be used to optimize the mechanical properties of AlSi10Mg alloys and potentially other aluminum alloys produced through additive manufacturing. The results of this study may provide not only fundamental insights into the grain refinement mechanism but also a promising paradigm for tailoring properties of AM Al-Si alloys.

## 2. Methodology

The samples used in this study were prepared using a TruPrint 1000 selective laser melting (SLM) system (Trumpf, Ditzingen, Germany) equipped with an ytterbium fiber laser. The following process parameters were applied to obtain 99.9% dense AlSi10Mg alloy samples [41]:

- Laser power = 175 W;
- Layer thickness = 20 µm;
- Scanning speed = 1400 mm/s;
- Scanning strategy—zigzag with 67° rotation;
- Build plate material—pure aluminum;
- Build plate temperature—room temperature.

To prevent contamination of the sample during fabrication, the substrate and scraper were positioned only after the powder was spread. Additionally, the start of the SLM process was postponed until the oxygen level in the chamber fell below 0.02%.

The commercially available AlSi10Mg alloy powder (Sigma Aldrich, Saint Louis, MO, USA) used in this study was spherical and had the following chemical composition: 87.8 Al, 10.5 Si, 0.5 Mg, 0.15 Ti, 0.15 Cu, 0.09 Fe in wt%, measured by energy dispersive spectroscopy (EDS).

The SLMed samples having a size of 15 × 15 × 60 mm were pre-machined to a size of 14.25 × 14.25 × 60 mm and then heat-treated at 320 °C for 9 min in a laboratory dryer under protective atmosphere. This preliminary step aimed to improve the workability of the samples and modify their microstructures as described in the previous study [32]. For clarity, the heat-treated sample is denominated “HT320” in this study.

Following heat treatment, the surface of the sample was covered with a thin layer of molybdenum disulfide (MoS_2_) to reduce friction between the sample and the ECAP die. Then, the sample was inserted into a custom-designed ECAP die with inner channel (Ψ) and outer (φ) angles of 90° and ~20°, respectively. Before initiating the deformation process, the billet was carefully inserted into the ECAP die. To ensure uniform temperature across the billet, it was preheated to the designated deformation temperature. To monitor and verify the billet temperature during the preheating phase, a thermocouple was inserted into the billet. The thermocouple placement was chosen to accurately represent the core temperature of the billet, minimizing any temperature gradients.

After preheating, the billet was pressed once through the ECAP die at 100 °C. For the purpose of clarity, the ECAP-processed sample is denominated as “HT320E100”. One ECAP pass corresponded to the equivalent von Misses plastic strain (ε_N_) of ~1, according to the following formula:

(1) $$\epsilon_{N}=\frac{N}{\sqrt{3}}\left[2cot\left(\frac{\varphi }{2}+\frac{\psi }{2}\right)+\psi csc\left(\frac{\varphi }{2}+\frac{\psi }{2}\right)\right]$$

In this equation, N equals to the number of ECAP cycles.

To analyze the microstructural changes induced by heat treatment and subsequent ECAP processing, we prepared metallographic samples. The samples were cut perpendicular to the built direction (HT320 sample) and extrusion direction (HT320E100 sample) to ensure a comprehensive and comparative evaluation of microstructure evolution.

The cut samples underwent a series of grinding and polishing steps to achieve a smooth (scratch-free) plain surface suitable for detailed microscopic examinations. Initially, the samples were ground using #800 and #1200 grit SiC water papers, which aimed to remove surface irregularities produced during cutting. Following the grinding phase, the samples were polished with diamond pastes of decreasing particle sizes (9–1 μm). To achieve the finest level of polishing (necessary for electron backscatter diffraction analysis), the samples were finally polished using a colloidal suspension of aluminum oxide (Al_2_O_3_) for 1 h.

To reveal the microstructure details samples were etched with a solution of 190 mL of distilled water, 2 mL of hydrofluoric acid, 3 mL of hydrochloric acid, and 5 mL of nitric acid for 10–20 s (Keller’s reagent).

Microstructural investigation was carried out using a Zeiss Supra 35 (Carl Zeiss NTS GmbH, Oberkochen, Germany) scanning electron microscope with an EDS detector and TSL-OIM system for EBSD and Transmission Kikuchi Diffraction (TKD) measurements. EBSD measurements were made using a 20 keV acceleration voltage, a specimen tilt angle of 72°, and a working distance of 17 mm. A step size of 0.04 μm was used for the finest EBSD scans.

After the scans were performed, the EBSD data were analyzed using TSL-OIM 7 (Edax, Mahwah, NJ, USA) and ATEX software version 4.14 (Universite de Lorraine, Metz, France) [43]. First, a routine clean-up procedure was performed, which consisted of a standardization of the grain confidence index and a subsequent correlation of the neighboring orientation under the following conditions: a grain misorientation of 5° and a minimum confidence index of 0.1. Afterward, grains with less than 9 pixels were excluded from the statistical analysis. A critical misorientation angle of 15° was used to distinguish between low-angle boundaries (LABs) and high-angle boundaries (HABs).

During the calculation of the average grain size using the linear intercept method, consideration was given to crystallite misorientations exceeding 5°. The Kernel Average Misorientation (KAM) maps were used to visualize and quantify the local misorientation distribution within the range of 0° to 5°. This range is often associated with the distribution of geometrically necessary dislocations. Furthermore, the grain reference orientation deviation (GROD), which measures the difference in orientation between a specific point and the average grain orientation, was used to highlight localized microstructural variations.

For the characterization of microstructures using transmission electron microscopy (TEM), lamellae measuring approximately 8 × 8 µm were prepared through focused ion beam (FIB) cutting. These samples were subsequently analyzed using a scanning transmission electron microscope (S/TEM) TITAN 80–300 (FEI Company, Hillsboro, OR, USA) operating at 200 kV. The TEM lamellae were cut parallel to the build and extrusion directions. To achieve improved spatial resolution in microstructure analysis, the transmission Kikuchi Diffraction (TKD) method was employed. The TEM lamellae were mounted on a custom-made holder, positioned at a net angle of 10° relative to the horizontal. The working distance was set at 7.5 mm, and the electron beam energy remained constant at 20 kV throughout the analysis. A suitable area within the thinnest part of the sample was selected for TKD, with data acquired using a step size of 20 nm. Subsequently, the TKD data underwent routine cleaning procedures, replacing non-indexed pixels with adjacent, indexed values—first with 6 indexed neighbors and then 5 indexed neighbors.

## 3. Results

### 3.1. Microstructure Analysis

First, the microstructure of the SLMed AlSi10Mg alloy was characterized in detail prior to ECAP processing. This part of the research aimed to clarify the importance of a unique microstructure in the grain refinement process under severe plastic deformation conditions.

**Figure 1:**
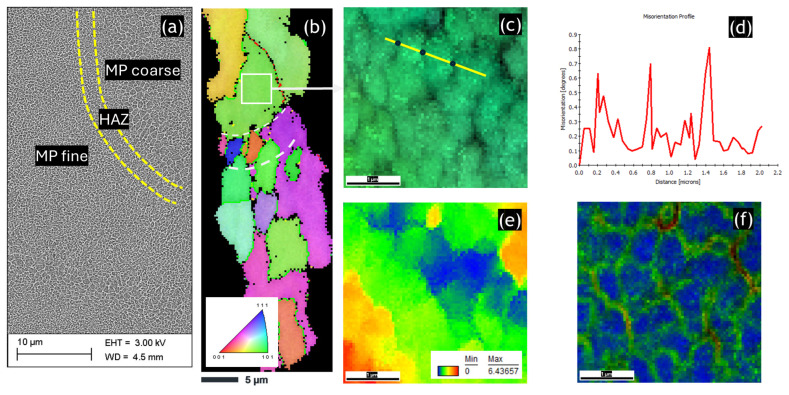
Microstructure of the SLMed AlSi10Mg alloy in HT320 condition: (**a**) Secondary electron image revealing the unique heterogeneous microstructure; (**b**) coarse IPF-Z EBSD map in which the red line corresponds to the LABs and the green line corresponds to HABs; (**c**) IPF-Z map from the white square area in (**b**) acquired with a step size of 40 nm; (**d**) point-to-point misorientation profile corresponding to the yellow line in (**c**). Peaks correspond to the Al/Si cell boundaries, which are additionally distinguished by black circles in the yellow line, (**e**) the GROD map, (**f**) the corresponding KAM map. The KAM distribution is visualized using a color-coded map, with the scale with scale varying from blue (misorientation between 0° and 1°), green (misorientation between 1° and 2°), yellow (misorientation between 2° and 3°), orange (misorientation between 3° and 4°) to red (misorientation between 4° and 5°).

Figure 1 shows the microstructures of the SLMed sample in the HT320 condition. As depicted in Figure 1a, the metastable Al/Si cells have been altered by heat treatment, resulting in a partially continuous Al/Si network in the HT320 sample. Additionally, three distinct zones are visible: the melt pool fine (MP-fine) zone, the melt pool coarse (MP-coarse) zone, and the heat-affected zone (HAZ). Each of these zones is characterized by different sizes and morphologies of the Si-phase/Al cells. In the MP-fine zone, the cells are 100–200 nm in size, while in the MP-coarse zone, the cells are about 400 nm in size. Notably, the HAZ zone does not exhibit a cellular morphology; instead, only isolated Si particles are visible in this area.

In Figure 1b, the heterogeneous grain size distribution is evident. The HAZ zone, indicated by the white dashed line, exhibits the smallest grain size due to the reheating cycle during the melting of the subsequent layer in the SLM process. This zone consists of grains approximately 3–5 μm in size. In contrast, the area surrounding the HAZ zone contains equiaxed grains ranging from 10 to 30 μm in size. Additionally, the analyzed area is predominantly characterized by high-angle boundaries, which comprise about 95% of the total boundary fraction.

**Figure 2:**
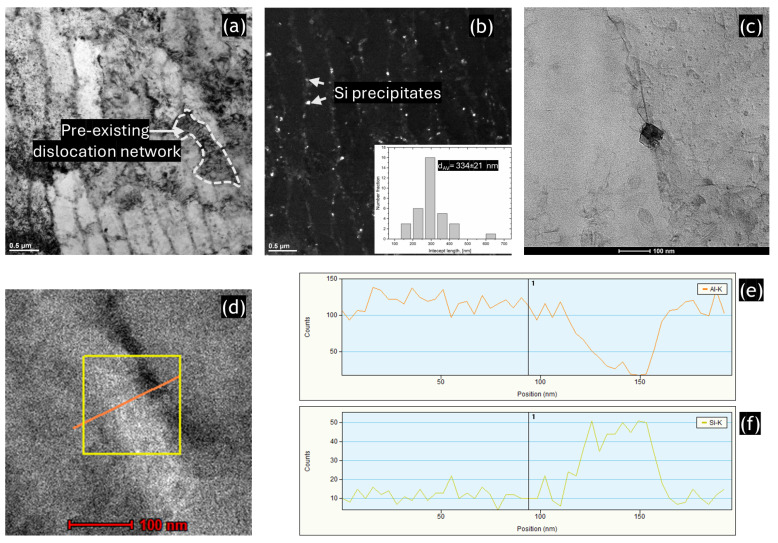
The results of TEM analysis of the SLMed AlSi10Mg alloy in HT320 condition: (**a**) Bright-field TEM image, (**b**) corresponding dark-field TEM image, and (**c**) STEM image of the Al/Si cell wall. It should be noted that the TEM sample was FIB cut along the building direction (as indicated by a white arrow), (**d**) STEM image presenting the area of the EDS profile measurement, (**e**) EDS profile of aluminum measured along the orange line in (**d**), (**f**) EDS profile of Silicone measured along the orange line in (**d**).

Figure 1c shows the IPF-Z image of the area marked by the white square box in Figure 1b. This image overlays the IPF color coding with the band contrast map, clearly distinguishing the Al/Si cells. Due to the high magnification, all observed cells are aligned with the [111] plane of a single equiaxed grain. As can be seen, the cell size ranges from 0.2 to 0.4 μm. This observation is consistent with the cell size seen in the SEM image and has been reported by other researchers [34,44]. The point-to-point misorientation profile, Figure 1d, which corresponds to the yellow straight line in Figure 2c, provides direct evidence of increased local misorientation at the cell boundary of 0.6–0.8°. It is worth noting that our observations are consistent with numerous studies [45,46], showing a misorientation of about 0.6° in SLM alloys.

To analyze misorientation and rotation gradients, KAM and GROD maps were plotted. The GROD map (Figure 1e) reveals a noticeable intragranular misorientation spread, likely due to the presence of organized dislocation structures (pre-existing dislocations resulting from the high cooling rate in the L-PBF process). The maximum observed intragranular misorientation is ~6.5° in the HT320 sample. High intragranular orientation differences are typically associated with incremental crystal lattice rotations, which are accommodated by GNDs. Accordingly, the GROD map confirms pronounced rotations across the Al/Si cell walls (i.e., cell interior to wall) within the same grain.

The KAM map (Figure 1f) confirms an extra storage of GNDs near the Al/Si cell boundaries. From the comparison of the IPF map in Figure 1c and the KAM map in Figure 1f, it is clear that these GNDs accommodate local crystal lattice misorientations existing between individual Al/Si cells.

Figure 2 presents the results of the TEM analysis of the HT320 sample. The bright-field TEM image in Figure 2a reveals elongated Al/Si cells. Using the line intercept method, we measured their width to be approximately 334 μm, which corresponds well with the cell size observed in the SEM images. The dark-field image clearly shows different contrasts between the Al cell interior and the Si cell wall, confirming their distinct orientations, Figure 2b. A higher magnification STEM image, along with corresponding EDS spectra, provides detailed information about the chemical composition of the nanosized precipitates visible along the cellular walls, as shown in Figure 2c–f. The TEM results provide evidence of the rupture and modification of cell walls after heat treatment, consistent with findings documented in several studies [47,48,49].

**Figure 3:**
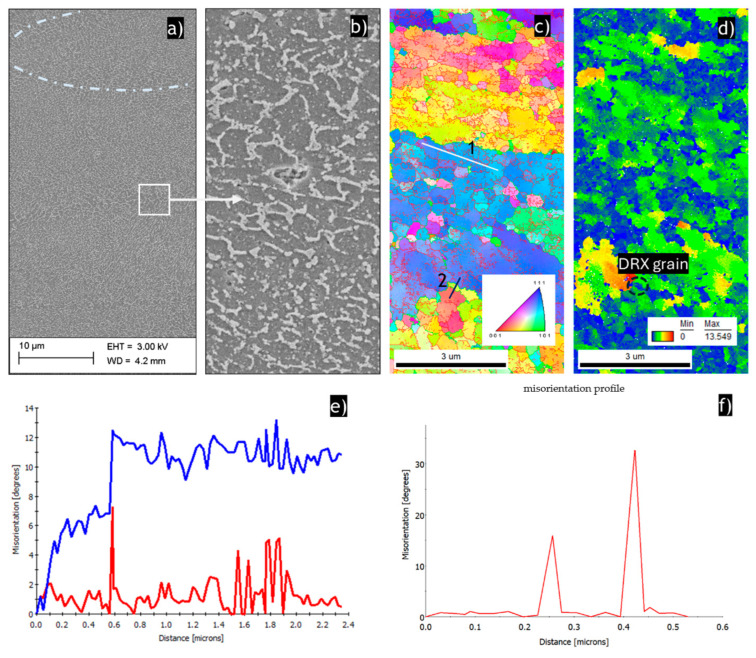
Microstructure of the SLMed AlSi10Mg alloy in HT320E100 condition: (**a**) Secondary electron image revealing the evolution of unique heterogeneous microstructure. It should be noted here that the microstructure evolved during ECAP processing; therefore, the discontinuous laser scan traces (observed on the build plane) were replaced by semicircular melt pools, which was caused by the unique shear characteristic in the ECAP process; (**b**) high-magnification SEM image; (**c**) IPF-Z map; (**d**) GROD map; (**e**) point-to-point (red) and point-to-origin (blue) misorientation profiles corresponding with the white (1) line in (**c**). The peak corresponds to the dislocation of cell boundaries that evolved from the Al/Si cellular structure. The misorientation analysis indicates that subgrain boundaries are formed in the vicinity of larger grains by crystal lattice rotations, (**f**) point-to-point misorientation profile corresponding to the black line (2) in (**c**).

Figure 3 presents the microstructures of the SLMed sample in the HT320E100 condition. As shown in Figure 3a, the unique L-PBF microstructure has not disappeared after ECAP processing, as there are clearly distinguishable typical features of the L-PBF microstructure such as semicircular melt pools along heat-affected zones (white dashed line). However, one can see that the metastable cellular structure has been significantly modified. Figure 3b clearly shows that the Si cell boundary breaks and coarsens after ECAP processing at 100 °C. According to the study by Al-Zubaydi et al. [39], during SPD processing, the eutectic silicon is significantly sheared, leading to the refinement of the Si phase. Here, particle refinement is particularly seen in the HAZ zone, where isolated particles existed before the ECAP procedure was applied.

Figure 3c presents the results of the EBSD analysis. The typical feature of the HT320E100 sample microstructure is submicrometric grains with average grain sizes of approximately 500 nm. The IPF-Z orientation map reveals average misorientation and LAGB fraction values of 11.5° and 78.7%, respectively. These low average misorientation values and high LAB fractions indicate the formation and rearrangement of dislocation boundaries during ECAP processing [50]. The point-to-point misorientation profile measured across several dislocation cells reveals an average misorientation of approximately 1.2°, and several peaks reaching 8°, Figure 3e. It can be seen that the distance between the peaks is approximately 300 nm. This implies that the dislocation cell boundaries have formed from the Al/Si cells, whose misorientation gradually increased after ECAP processing because of the continuous crystal lattice rotations of Al/Si cells. Furthermore, it is revealed that ultrafine dynamically recrystallized grains (DRX) with misorientation >15° formed within the microstructure, as shown in Figure 3f.

Next, local misorientations and crystal lattice rotation gradients were analyzed using the grain reference orientation deviation (GROD) function. The map in Figure 3d shows GROD values ranging from 0° to 13.5°, reflecting significant deformation heterogeneity, with the highest GROD values corresponding to areas of high dislocation density [51]. In this map, grains with low GROD values (blue grains with low internal misorientation) can be considered DRX grains [52].

**Figure 4:**
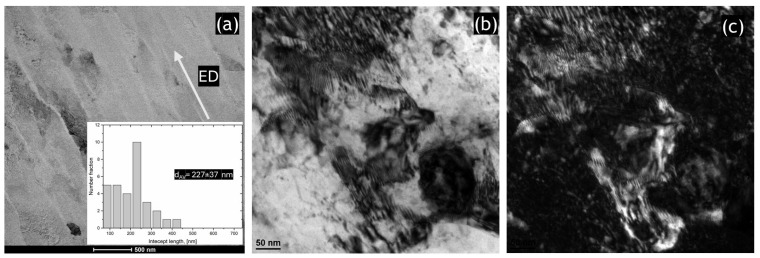
Results of TEM analysis of the SLMed AlSi10Mg alloy in HT320E100 condition: (**a**) Low-magnification STEM image revealing the parallel boundaries, (**b**) the high-magnification bright-field TEM image, (**c**) the corresponding dark-field TEM image. It should be noted that TEM sample was taken along the extrusion direction (TD-ED).

Dislocation activation and the grain refinement process were further analyzed via TEM. Figure 4a shows the STEM image of the HT320E100 sample. After a single ECAP pass, the microstructure was characterized by a substantial increase in dislocation density and the emergence of a banded (lamellar) structure with nearly parallel boundaries appeared. It should be noted that dislocation walls exist inside elongated grains, forming smaller subgrains that subdivide the lamellar Al/Si cells. Using the line intercept method, we measured its width to be approximately ~227 μm, which corresponds well to the SEM/EBSD observations.

TEM analysis of the microstructure reveals strong local strain contrasts due to the high density of dislocations introduced at 100 °C. Bright-field and dark-field TEM images (Figure 4b,c) show these dislocations forming dense dislocation walls (DDWs), which constitute the observed low-angle grain boundaries (LABs). These DDWs are identified as GNBs, a type of deformation-induced boundary, typical of ECAP-produced aluminum alloys where dynamic recovery is the primary softening mechanism [53,54].

As evidenced, grain refinement involves the formation and evolution of subgrain structures. Initially, GNBs form, dividing coarse grains into smaller cell blocks. In addition, Incidental Dislocation Boundaries (IDBs) can develop due to the statistical trapping of glide dislocations, further subdividing the grains. Increased plastic strain leads to an increase in misorientation between individual cells, driven by dislocations moving on different slip systems, resulting in the transformation of low-angle grain boundaries into high-angle grain boundaries. This process creates a finer, more complex grain structure with higher dislocation densities and enhanced material properties.

**Figure 5:**
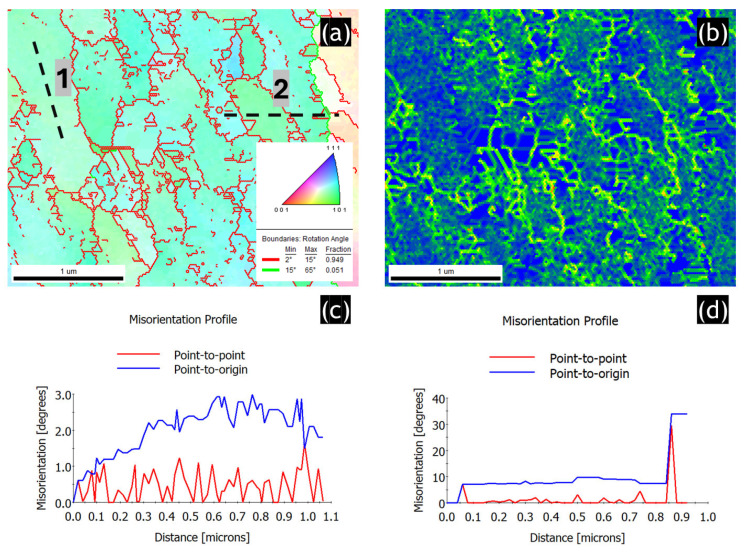
Results of EBSD-TKD analysis of the SLMed AlSi10Mg alloy in HT320E100 condition: (**a**) IPF-Z image, (**b**) The corresponding KAM map. KAM distribution is represented in a color-coded map with scale varying from blue (misorientation between 0° and 1°), green (misorientation between 1° and 2°), yellow (misorientation between 2° and 3°), orange (misorientation between 3° and 4°) to red (misorientation between 4° and 5°), (**c**) point-to-point misorientation profile corresponding to the black line (1) in (**a**), (**d**) point-to-point misorientation profile corresponding to the black line (2) in (**a**).

The TKD technique was employed to analyze the grain microstructure of TEM lamellae with a higher spatial resolution; Figure 5. Figure 5a shows the IPF-Z image in which the LAB network is clearly seen. As shown, the LAB network represents about 95% of the total grain boundary fraction. To analyze the distribution of dislocations at the nanoscale, the KAM map was generated; Figure 5b. It is evident that regions with a high density of grain boundaries (GNDs) are concentrated around grain boundaries (GBs). This is due to the strain partitioning between the soft Al and hard Si cell boundaries during plastic deformation. These GNDs were generated to accommodate local shape or orientation changes.

To investigate the local orientation changes accommodated by the GNDs, point-to-point misorientation profiles were plotted. The first profile, shown in Figure 5c, was taken along the extrusion direction to measure the misorientation within the elongated grain. It reveals that the orientation is almost constant along the length of the grain. The point-to-point misorientation curve shows only small misorientation variations (less than 1.5°) in the grain interior, implying the presence of dislocation structures. This is consistent with the TEM results showing multiple DDWs. The second profile, in Figure 5d, was taken perpendicularly to the extrusion direction from the area where higher KAM values were detected. It shows that the lattice orientation changes significantly at the Al/Si cell boundary. More specifically, lamellar grains visible in STEM images are separated mainly by LABs whose misorientation is about 5–6°. The point-to-origin profile that represents the lattice orientation changes relative to the first point reveals that misorientation does not accumulate across several low-angle grain boundaries but instead exhibits an oscillating behavior upon reaching HAB with a misorientation of about 30° (see a peak at the distance of about 0.9 micrometers).

### 3.2. Grain Refinement Model

**Figure 6:**
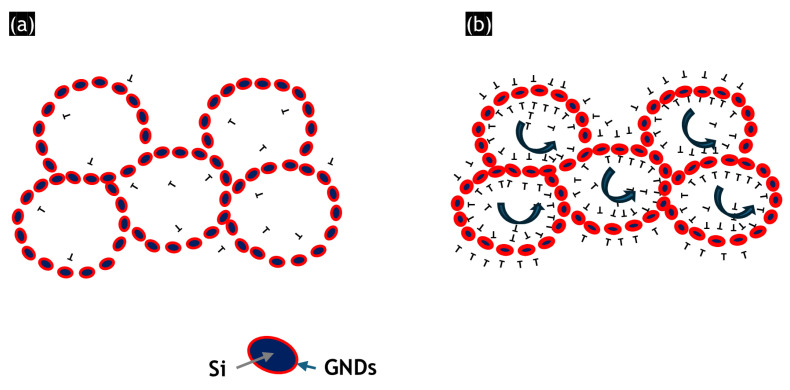
A schema illustrates the grain refinement of the ECAP processed AlSi10Mg alloy: (**a**) as-built condition, (**b**) after 1 ECAP pass at 100 °C. Note that the image schematically represents the equiaxed cells that were observed on the build and extrusion plane (Figure 2a and Figure 4a, respectively).

The grain refinement occurring during ECAP processing can be illustrated by the model, as depicted in the schematic diagram in Figure 6.

From the experimental data presented in this article, it is clear that pre-existing dislocations were present in the AlSi10Mg alloy. In addition, a detailed EBSD analysis revealed an accumulation of GND near the cell boundary area. Furthermore, it was revealed that the average misorientation between Al/Si cells is very low ~0.6°. Such a microstructure is schematically drawn in the image on the left.

According to the experimental results, after one ECAP pass, a significant number of statistically stored dislocations (SSDs) accumulated in the material. The movement of most of these dislocations was hindered by the Si cellular boundaries. As a result, dense dislocation walls formed, manifesting as low-angle boundaries (LABs) within the grains, as evidenced by the EBSD data. Additionally, a substantial number of geometrically necessary dislocations (GNDs) were generated near the heterointerfaces, particularly at the Al/Si interfaces, to accommodate incompatible deformation. These GNDs acted as barriers to the movement of the newly formed SSDs, thereby contributing to the refinement of the grain structure [55]. In general, the accumulation of dislocations resulted in the formation of dislocation cells in the form of intragranular LAB (due to the dynamic recovery) [56], thus increasing the fraction of LABs in the microstructure. Moreover, the TKD analysis showed that the grains rotated significantly due to a large accumulation of strain. This caused an increase in lattice misorientation between individual cells. In other words, after deformation, the cells rotated, leading to greater misorientation, as evidenced by the TKD analysis. Furthermore, some of these LABs are converted into HAGBs to generate the dynamically recrystallized grains, as revealed in Figure 3.

## 4. Conclusions

In conclusion, the microstructure evolution of the AlSi10Mg alloy subjected to one ECAP pass was analyzed in detail using electron microscopy. The main findings are as follows:

- The unique heterogeneous microstructure was preserved after heat treatment. Electron microscopy analysis revealed a multiscale heterogeneous microstructure composed of bimodal grains, melt pool structures, and Al/Si cells.
- Intensive accumulation of dislocations resulted in a refinement of the grain structure down to the sub-micrometer scale. After one ECAP pass, the average grain size of the grains attained ~500 nm.
- The Al/Si cell boundaries significantly contributed to grain refinement, mainly because of their tendency to hinder dislocation movement.
- High-resolution TKD results demonstrated that the Al/Si cellular wall lattice orientation increased after ECAP processing. The newly formed subgrain boundaries have misorientation between 2 and 8°, thus contributing to grain refinement.

## Data Availability

Data available on request.
